# Comparative study on the role of extracts of male and female of jojoba’s leaves in the green synthesis of α-MnO_2_ as an antimicrobial agent

**DOI:** 10.1038/s41598-026-65815-8

**Published:** 2026-08-27

**Authors:** Somia M. Abbas, Rasha S. El-Tawil, Hanaa M. Abuzeid, Ashraf E. Abdel-Ghany, Mohamed S. Abdel-Aziz, Ahmed M. Hashem

**Affiliations:** 1 Inorganic Chemistry Department, National Research Centre, 33 El Bohouth St., (Former El Tahrir St.), Dokki, Giza, 12622 Egypt; 2 Microbial Chemistry Department, Biotechnology Research Institute, National Research Centre, 33 El Bohouth St., (Former El Tahrir St.), Dokki, Giza, 12622 Egypt

**Keywords:** Green synthesis, Fresh leaves of male and female jojoba’s leaves, M-MnO_2_, F-MnO_2_, Four microorganisms, Antimicrobial efficacy, Biological techniques, Biotechnology, Materials science, Microbiology, Nanoscience and technology

## Abstract

The green synthesis method is a predominant trend as it is an ecofriendly, cleaner, scalable, and cheaper method and viable to form biocompatible NPsrather than the traditional methods. Jojoba plant is a natural and strong antioxidant as it contains phenolic and flavonoids compounds.Since male and female jojoba plants are known to differ in enzymatic and non-enzymatic antioxidant activity and phytochemical composition, this comparison was designed to test whether such sex-linked phytochemical variability translates into measurable differences in the structural, morphological, and antimicrobial properties of the resulting MnO_2_ nanoparticles. This study focuses on preparation of MnO_2_ nanoparticles using the extract of male (M-MnO_2_) and female (F-MnO_2_) jojoba^’^s leavesand to evaluate theirantimicrobialactivity.The prepared samples were characterized using X-ray diffraction (XRD), energy dispersive X-ray (EDX) and Thermal analyses (TGA & DTG) to assess their structural and physicochemical properties. The scanning electron microscopy (SEM) and transmission electron microscopy (TEM) also used to assess the morphology and microstructure evolution. The X-ray diffraction analysis confirmed the cryptomelane-type α-MnO_2_ phase for both samples distinguished by its characteristic (2 × 2) tunnel framework capable of hosting potassium ions within the crystal lattice. The microstructure analyses also reveal the nanocrystalline nature with flake morphology of MnO_2_ NPs that enhances the surface reactivity helping in improving the antimicrobial performance.The antimicrobial efficacy of the two samples (M-MnO_2_ and F-MnO_2_) was evaluated throughtheCup Diffusion Agar Method and the Colony Forming Unit (CFU) Assay using Four representative microorganisms were selected for testing: Staphylococcus aureus ATCC 6538-P (Gram-positive bacterium), Escherichia coli ATCC 25,933 (Gram-negative bacterium), Candida albicans ATCC 10,231 (yeast), and Aspergillusniger NRRL-A326 (filamentous fungus).The findings of this research highlight sample F-MnO_2_ as a potential antimicrobial agent with higher percent of the inhibition zones and higher reduction percent of the number of colonies formation.

## Introduction

MnO_2_ is an outstanding metal oxide which has a wide spectrum of applications^1,2^ such as, inorganic pigment within ceramic matrices, an electrode material in energy storage and conversion devices e.g. rechargeable batteries and solar cells^3,4^, an active component in supercapacitors^5^, a photocatalytic agent^6^, a medium for selective ion and molecular separations^7^, a sorbent for the remediation of toxic metal contaminants^8^, a depolarizer in traditional Leclanché cells^9^ and as a catalyst in various chemical processes^10^.

Various synthesis methods have been used to prepare MnO_2_ with particular structures, morphologies, and sizes for practical production or tests^11–13^. These methods include the hydrothermal method^14^, sol–gel approach^15^, template method (soft & hard)^16^, electrodeposition method^17^, reflux approach^18^ microemulsion approach^19^, chemical co-precipitation^20^ and reduction method^21^.The reduction of permanganate (Mn(VII)) can be achieved through the utilization of reducing agents like sodium hydroxide (NaOH), ammonium fluoride (NH_4_F), nitric acid (HNO_3_), and hydrochloric acid (HCl) via various reaction pathways^22,23^. The chemical synthesis use toxic, hazardous, and costly chemical reagents for reduction and surface capping processes may pose a risk to environmental sustainability. Furthermore, physical synthesis methods typically necessitate sophisticated and costly instrumentation, demanding highly skilled operation and often involving time-consuming and energy-intensive procedures^24,25^.

The green synthesis method using the natural antioxidant of polyphenols in plant extracts as reducing agents can mitigate the reliance on hazardous chemicals and circumvent the need for advanced technological equipment.Using the extract of plants is a predominant trend as it is an ecofriendly, cleaner, scalable, and cheaper method and viable to form biocompatible NPs^26,27^. The structural mechanism generally proposed for this type of biogenic reduction is primarily driven by polyphenols and flavonoids present in plant extracts^54,55,93^. The hydroxyl (-OH) groups present on these antioxidant rings readily form coordinate complexes with high-valence manganese precursors (e.g., permanganate, Mn(VII). This is followed by an intermolecular electron transfer that reduces Mn(VII) to the stable Mn(IV) state, precipitating out as MnO_2_ nuclei while the polyphenols are concurrently oxidized into quinone derivatives^55,56^. The residual biomolecules remain adsorbed onto the newly formed nanoparticle (NP) surfaces, providing steric stabilization that hinders particle agglomeration without the need for synthetic polymers^56,57^. This mechanism describes the general pathway established for polyphenol-mediated green synthesis of metal-oxide nanoparticles; the specific phytochemical composition of the male and female jojoba extracts used in this study was not independently characterized “(section 3.4)” green synthesis nanoparticles specifically remains an active area of research, with recent studies continuing to report new plant-extract-mediated routes and their functional characterization^94^. Jojoba plants an economicallyimportant antioxidant flavonoid compound which can be used in many disorders treatment such as asthma, inflammation, and cancer; besides its antimicrobial, antiproliferative and antifungal activities^28^. Jojoba, *Simmondsiachinensis*(Link) Schneider is a commercially importantdioecious industrial cropevergreendesertshrub with male and female flowers (20–50 cm in height) that can grow to a height of 3 m in dry areas^29^. Native Americans use this plant as a traditional medicine for infectious diseases, cancer, colds, dysuria, obesity, postpartum, sore throat, and sores^30,31^. On the other hand, jojoba^’^s leaveswere reported as eco-friendly detoxicant^32^.

In Egypt, jojoba is considered as one of the most practical solutions for desert plantation because its ability for tolerance to heat, drought and salt, lesser possibilities for infection, lesser need for fertilizers, and generous financial income, are certainly the most encouraging goals to this plant in Egypt for revegetation of arid areas as it can survive in harsh desert environments^33^. *Simmondsiachinensis* was introduced to Egypt in 1991, with initial cultivation efforts utilizing seeds sourced from the United States and implemented under the environmental conditions of the Middle Sinai region. Cultivation of *Simmondsiachinensis* commenced in the Beheira Governorate of Egypt in 2017, with the initial seed harvest occurring in 2019, following a two-year cultivation period^34,35^.

Jojoba plant contains phenolic compounds, phytosterols, toccopherols, flavonoids and simmondsin and its products: simmondsin-3′-ferulate, 4, 5-didemethylsimmondsin and 4-demethylsimmondsin-2′-ferulate which have antioxidant activity^36–42^.These flavonoids include quercetin 3, 3ˋ-dimethyl ether, isokaempferide and quercetin 3-methylether^43^. The natural antioxidant activity and the oxidative stability of jojoba plant depends on the phenolic and flavonoid compounds present in the plant which differs according to the plant parts (seeds and leaves extracts). Yeşim KAR^40^ reported that the highest level of the phenolic compounds expressed as gallic acid equivalent (GAE) was found in S. chinensis, leaf (jojoba leaf: 313 mg/g GAE), conversely, the lowest was in S. chinensis seed (jojoba seed:~120 mg/g GAE). The higher phenolic component means the higher antioxidant properties and higher Free radical scavenging activity.

Jatin Kumar and Jameel R. Al-Obaidi^29,44^ reported that jojoba males showed higher activities of enzymatic and non-enzymatic antioxidants. As males showed elevated levels of protein, proline, cysteine and higher activities of catalase, guaiacol peroxidase, glutathione reductase, ascorbate peroxidase and superoxide dismutase compared to females.Abdel-Mageedet.al^45^ succeeded to isolate 10 flavonoids and 4 lignans from the leaf extracts of S. chinensisand exhibited strong antioxidant and lipoxygenase activities.

The paucity of prior studies on the activity of extract of jojoba^’^s leave^29,46^ has motivated us to investigate its active constituents for synthesis of MnO_2_ NPs and its potential biological activities. Infections caused by multidrug-resistant (MDR) microbes are responsible for an estimated 700,000 deaths annually, a number projected to rise to 10 million by 2050 if no effective interventions are implemented^47^. In response to this challenge, nanotechnology offers a promising alternative, particularly in the form of metal oxide nanoparticles. Among them, manganese dioxide (MnO_2_) nanoparticles have attracted considerable attention due to their advantageous physicochemical characteristics, including large surface area, high redox potential, and their ability to generate reactive oxygen species (ROS)^48^. These features endow MnO_2_ nanoparticles with potent and broad-spectrum antibacterial activity, effective against both Gram-positive and Gram-negative bacteria. Furthermore, they have shown promising utility in diverse applications such as wound healing, water disinfection, and biomedical device coatings^48^.

The present study aims to synthesize MnO_2_ nanoparticles using *Simmondsia chinensis* leaf extracts through an environmentally friendly green synthesis approach and evaluate their antibacterial activity. Beyond developing a sustainable route for MnO_2_ production, the study investigates whether the naturally occurring phytochemical differences between male and female joujoba plants can be exploited as a performance of the tailor the structural characteristic and antimicrobial performance of the resulting nanoparticles. The findings are expected to contribute the growing field of sustainable nanotechnology and support the development of novel antimicrobial materials for combating drug-resistant bacterial infections.

A key aspect of the present work is the comparison between male and *Simmondsia chinensis* leaf extracts. Jojoba is dioecious species, and previous phytochemical and physiological and physiological studies have demonstrated systematicdifferences between male and female planets in enzymatic and non-enzymatic antioxidant activity, as well as in protein and proline content^29,44^. In addition, leaf extracts of *S. chinensis*, although not previously compared on the basis of plant sex, have been reported to contain diverse flavonoids and lignans with significant antioxidant properties^45^. Such phytochemical differences may influence the reduction kinetics of metal precursors, nucleation behavior, crystal growth, and the incorporation of structural defects or guest ions during nanoparticle formation.

Although this compositional variability has been well established in plant-physiology, it has not previously been exploited as a controlled design study employs mal and female jojoba leaf extracts as two naturally occurring yet compositionally distinct bio-reducing agents while maintaining identical synthesis conditions through the preparation process. This experimental design minimizes the influence of external synthesis parameters, allowing the observed differences in MnO_2_ phase purity, crystallinity, potassium incorporation and antimicrobial potency  (Sections 3.1-3.4) to be attributed, as far as possible, to intrinsic phytochemical characteristic of the reducing agents.

To the best of our knowledge, this study presents the first structure-composition-activity comparison of MnO_2_ nanoparticles synthesized using sex-differentiated extract from the same planet species. Unlike previous green- synthesis studies, which primarily compared different plant species or different synthesis routes, the present approach isolates plant sex as principal experimental variable, thereby providing new insight into how naturally occurring phytochemical diversity can be utilized to tailor the structural and functional properties of green-synthesized metal-oxide nanomaterial.

## Materials and methods

### Materials

All chemicals were of analytical grade and were used as received, without any additional purification. Deionized water was utilized for preparing all solutions. Potassium permanganate (KMnO_4_) served as the manganese precursor. Fresh leaves of cultivated male and female jojoba (*Simmondsia chinensis*) were collected in February 2025 from a private farm in Ismailia, Egypt, dried, and processed to obtain the plant extracts used in the green synthesis of MnO_2_ nanoparticles. The plant is commercially cultivated and was not collected from wild populations. Collection was conducted with the permission of the farm owner. The plant was identified based on morphological characteristics according to standard taxonomic descriptions. As the plant material was obtained from a cultivated commercial source and not from wild habitats, deposition of a voucher specimen was not required. All procedures complied with relevant institutional and national guideline.

### Formulation procedures for MnO_2_-nanoparticles

**Fig. 1:**
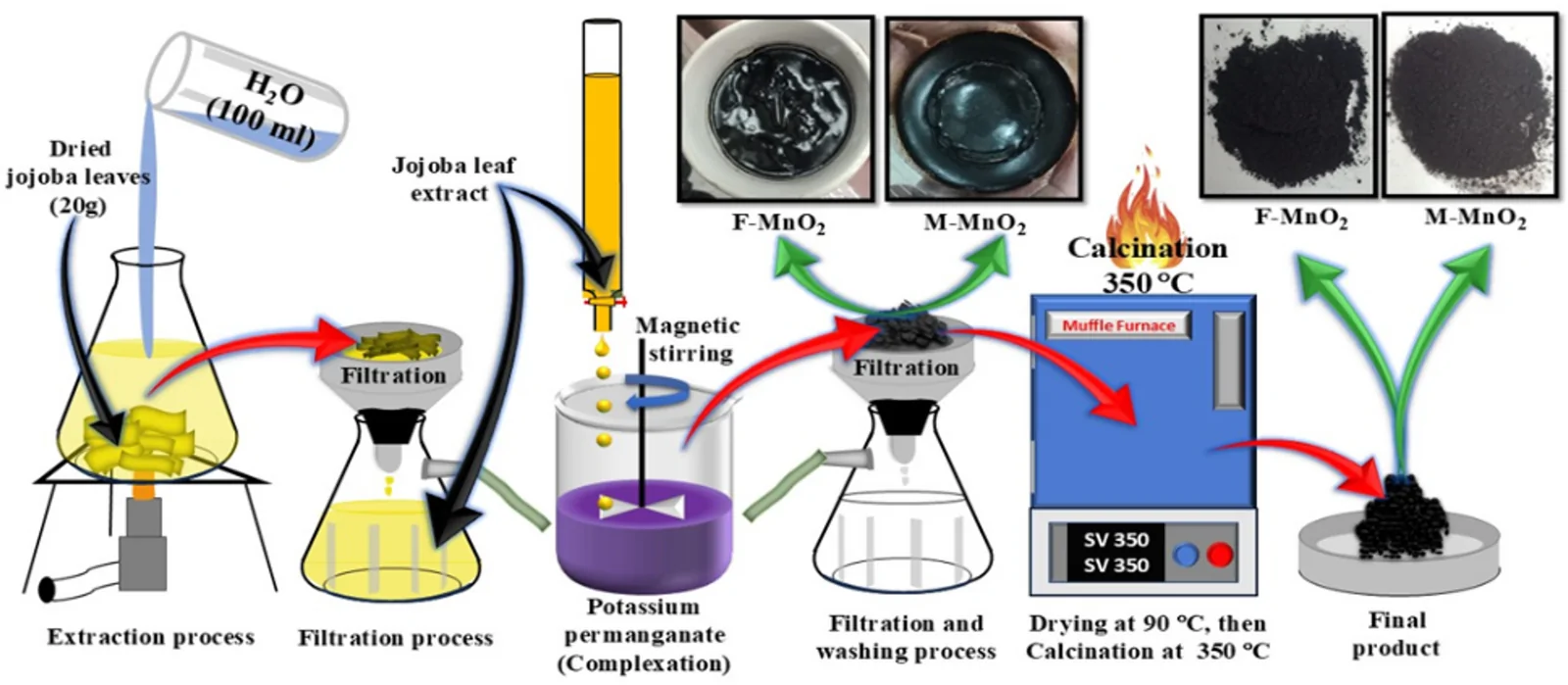
Schematic diagram for the synthesis of M-MnO_2_ and F-MnO_2_.

Male and female MnO_2_ were synthesized using a green chemical method as shown in Fig. 1. For each extract of Male and Female jojoba’s leaves, 20 g of dried male or female *Simmondsiachinensis* were boiled in 100 mL of distilled water at 100 °C for 15 min. After cooling to room temperature, the mixtures were filtered to obtain clear plant extracts. Separately, 3 g of KMnO_4_ was dissolved in 100 mL of deionized water. The corresponding extract (male or female) was then added dropwise to the KMnO_4_ solution under vigorous stirring for 1 h at room temperature. A visible color change from purple to black confirmed the reduction of KMnO_4_ to MnO_2_ by the plant-derived phytochemicals.

The resulting black precipitate was collected by filtration, thoroughly washed with distilled water to remove residual impurities, and dried at 90 °C overnight. The dried solids were subsequently calcined at 350 °C for 5 h to improve crystallinity and eliminate remaining organic components. This calcination temperature was selected based on the thermal behavior of the as-precipitated solids “ (Section 3.1)”: TGA/DTG showed that loss of physiosorbed water and residual organic/phytochemical matter is essentially complete below 300 °C, while decomposition of the MnO_2_ framework itself only begins above 500 °C. A calcination temperature of 350 °C for 5 h therefore removes adsorbed moisture and residual biomolecules while remaining well below the temperature at which the tunnelled α-MnO_2_ structure begins to decompose, consistent with calcination windows used for other biosynthesized MnO_2_ nanostructures^65,67^. The reaction pH was not directly monitored during synthesis; this is acknowledged as a methodological limitation, since reduction of KMnO_4_ by plant polyphenols is known to be pH-sensitive, and organic acids naturally present in the extracts are expected to render the reaction medium mildly acidic. This is consistent with the minor β-MnO_2_ contribution detected by Raman spectroscopy in M-MnO_2_ R.(Section 3.3) The MnO_2_ samples synthesized using male and female extracts were designated as M-MnO_2_ and F-MnO_2_, respectively.

### Preparation of the test samples

To thoroughly assess the antimicrobial activity of the test samples, two complementary methods were employed: the cup diffusion agar assay and the colony-forming unit (CFU) count. This combined approach enabled both spatial evaluation of inhibition zones and quantitative measurement of microbial viability. Four representative microorganisms were selected for testing: Staphylococcus aureus ATCC 6538-P (Gram-positive bacterium), Escherichia coli ATCC 25,933 (Gram-negative bacterium), Candida albicans ATCC 10,231 (yeast), and Aspergillusniger NRRL-A326 (filamentous fungus). These strains were chosen to encompass a broad spectrum of microbial categories, including bacteria, yeast, and fungi.Neomycin (100 mg/mL) and Cycloheximide (100 mg/mL)served as the positive control against the bacterial strains, yeast and fungal.Test samples were prepared by dissolving 50 mg of each compound in 2 mL of dimethyl sulfoxide (DMSO). An aliquot of 100 µL from each solution was used for both assays.

#### Cup diffusion agar method

For the agar diffusion assay, nutrient agar plates were used for bacterial and yeast cultures, while potato dextrose agar plates were employed for fungal testing. Each plate was inoculated with 0.1 mL of a microbial suspension containing approximately 10^5^–10^6^ cells/mL. Wells (6 mm diameter) were aseptically punched into the agar and filled with 100 µL of the test sample. To promote optimal diffusion of the compounds into the agar, the plates were pre-incubated at 4 °C for 2–4 h. Afterward, bacterial and yeast plates were incubated at 37 °C for 24 h, whereas fungal plates were incubated at 30 °C for 48 h in an upright position to ensure uniform growth. Antimicrobial activity was evaluated by measuring the diameter of the inhibition zones surrounding each well, expressed in millimeters (mm). Antimicrobial activity was evaluated in triplicate for each sample against each microorganism, and zone of inhibition diameters are reported as mean ± standard deviation.

#### Colony forming unit (CFU) assay

In parallel, a CFU assay was performed to quantify microbial growth inhibition in liquid media. Microbial suspensions (10 µL) containing approximately 10^7^ CFU were inoculated into 10 mL of freshly prepared nutrient broth (5 g/L peptone, 3 g/L beef extract, pH 6.8) in sterile 100 mL Erlenmeyer flasks. For fungal assays, potato dextrose broth was used in place of nutrient broth. Each test flask received 50 mg of the sample, whereas control flasks were inoculated without any added sample, and additional flasks containing Neomycin (100 mg/mL) or Cycloheximide (100 mg/mL) served as the positive controls for the bacterial and fungal/yeast cultures, respectively.All cultures were incubated for 24 h under appropriate conditions: bacterial cultures at 37 °C and fungal cultures at 28 °C. Microbial growth was initially assessed spectrophotometrically by measuring the absorbance of each culture. Following incubation, serial dilutions were plated onto agar, and colony-forming units (CFUs) were counted to evaluate microbial viability.The percentage reduction in colony count (R%) relative to untreated controls was calculated using the following equation:

$$R (\%)=\mathrm{A}-\mathrm{B}/\mathrm{A} \times 100.$$

Where A is the no. of colonies of the culture control but B is the no. colonies of treated sample^49^.

### Characterization

Characterization of the nanoparticles included X-ray diffraction (XRD) using a Philips X’Pertdiffractometer (CuKα radiation, λ = 1.54056 Å, 2θ range: 10°–80°). Thermogravimetric analysis (TGA) was performed on a PerkinElmer TGA 7 series instrument from 30 to 1000 °C in air at a heating rate of 10 °C min⁻¹. Raman spectra were recorded using a portable i-Raman Plus 532 S laser Raman spectrometer equipped with a BAC151C microscopic imaging system (B&W TEK, USA), with objectives ranging from 20-100X. Surface morphology and elemental composition were examined by scanning electron microscopy (SEM) coupled with energy-dispersive X-ray spectroscopy (EDS) using a JEOL JSM-6060 LVD microscope.The transmission electron microscopy (TEM) image was acquired using a JEOL JEM-2100 F microscope (Tokyo, Japan). This instrument was run at 200 kV and included a Cs corrector, allowing for atomic resolution better than 0.14 nm.

## Results and discussion

### Structural analyses

#### X-ray diffraction (XRD) analysis

The crystalline structure of manganese dioxide (MnO_2_) synthesized using extracts of jojoba’s leavesfrom male (M-MnO_2_) and female (F-MnO_2_) was characterized by X-ray diffraction (XRD), as illustrated inFigure2. Both bio-synthesized samples produced via the reduction of KMnO_4_ exhibited diffraction patterns consistent with the standard tetragonal α-MnO_2_ phase (JCPDS 44–0141, space group *I4/m*), confirming the formation of the characteristic (2 × 2) tunneled structure^5,50^. Prominent diffraction peaks observed at 2θ = 12.7°, 37.4°, and 65.4°, corresponding to the (110), (211), and (002) crystallographic planes, respectively, further validate the presence of the tetragonal α-MnO_2_ phase^51^. The absence of additional peaks associated with other MnO2 polymorphs indicates a high degree of phase purity in both samples.

Although the synthesized samples correspond to the reference structure, notable variations in peak intensity and broadening were observed. Both M-MnO_2_ and F-MnO_2_ samples exhibited significantly broader and less intense diffraction peaks, particularly at the (211), (301), and (310) reflections, suggesting reduced crystallinity and a partially amorphous character. Such features are commonly associated with bio-synthesized nanostructures due to their nanocrystalline nature^52,53^. Interestingly, the F-MnO_2_sample showed sharper and more intense peaks compared to M-MnO_2_, indicating a higher degree of crystallinity and a more ordered framework. Crystallite sizes estimated via the Scherrer equation(Table 1)were approximately 25 nm for F-MnO₂ and 15 nm for M-MnO_2_, underscoring the impact of synthesis conditions on the structural evolution.

**Fig. 2:**
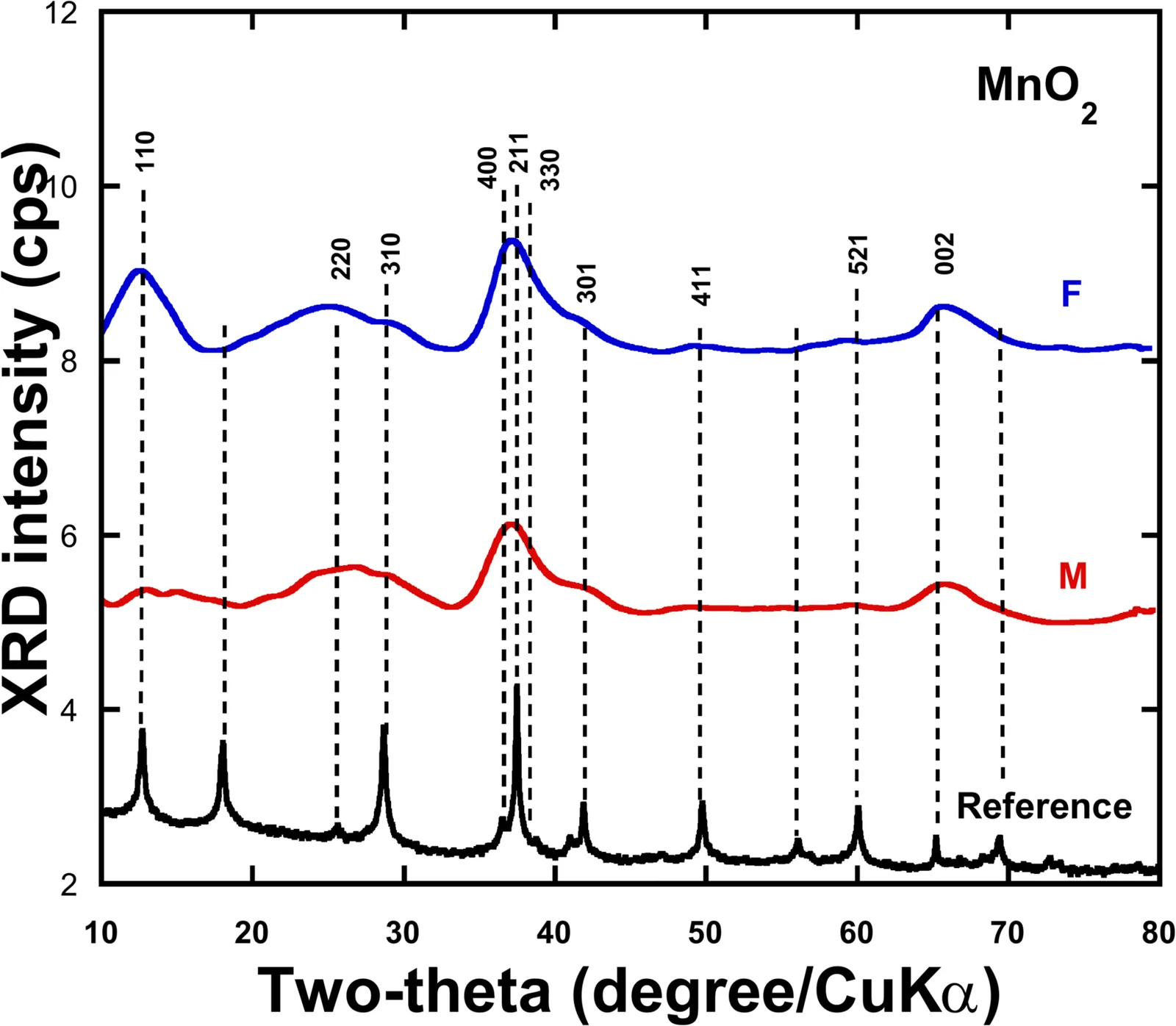
X-ray powder diffraction patterns of green-synthesized MnO_2_ using male (M) and female (F) Jojoba plant extracts.

Crystallite sizes reported in Table 1 were obtained directly from the Scherrer equation to the (211) reflection without a separate instrumental-broadening correction (e.g., from a NIST LaB6 or Si standard); the reported values should therefore be regarded as apparent, upper-bound crystallite sizes rather than absolute ones. Both samples were measured under identical instrumental conditions (same diffractometer, slit configuration, and scan parameters), so the relative difference between M-MnO_2_ and F-MnO_2_ is considered more reliable than either individual absolute value, and is not expected to be an artifact of uncorrected instrumental broadening. Likewise, a full Rietveld refinement and quantitative crystallinity index were not performed in the present work; the “high crystallinity” and “low crystallinity” descriptors used in the text refer qualitatively to the sharper, more intense reflections of F-MnO_2_ relative to M-MnO_2_ (Fig. 2) rather than to a numerically refined crystallinity percentage. Rietveld-based quantification of phase fractions and crystallinity is planned as part of a follow-up structural study.

These differences are likely due to variations in the phytochemical composition and concentration of bioactive compounds between the extracts of male and female jojoba’s leaves, which are known to affect the reduction kinetics and crystal growth during green synthesis^54,55^. Specifically, components such as polyphenols and flavonoids in the plant extracts serve dual roles as reducing and capping agents, thereby regulating the nucleation and growth of MnO_2_nanoparticles^56,57^. The higher concentration of these compounds in the female extract slows down the reduction kinetics, allowing for more controlled and homogeneous nucleation, which promotes the formation of a more ordered crystalline lattice. In contrast, the lower concentration in the male extract leads to faster, less controlled reduction, yielding smaller crystallites with lower overall crystallinity and a higher proportion of structural defects.Lattice parameters were determined through least-squares refinement using tetragonal system indexing (I4/m). The results (Table 1) closely matched reported literature values for α-MnO_2_^5,50,58^, showing minimal variation between the samples.

**Table 1:** XRD Parameters for MnO_2_ materials synthesized using male (M-MnO_2_) and female (F-MnO_2_) Jojoba extracts.

Sample	a (Å)	c (Å)	V (Å^3^)	Crystal size (nm)
F-MnO_2_	9.78 (8)	2.86 (5)	274.46	25
M-MnO_2_	9.79 (7)	2.85 (7)	274.20	15
Reference	9.78 (5)	2.86 (3)	274.12	–

The structural and compositional characteristics of the biosynthesized MnO_2_ nanomaterials were systematically analyzed to gain deeper insights into their formation mechanism and framework stability. In the α-MnO_2_ structure, potassium ions (K⁺),owing to their relatively large ionic radius (1.33 Å), preferentially occupy the 2e Wyckoff tunnel sites and provides the charge steric support needed to hold the (2 × 2) octahedral walls of the cryptomelane tunnel open^59^; without this support, the framework tend to collapse toward smaller-tunnel polymorphs such as (1 × 1) β-MnO_2_. During biogenic synthesis, the moderate availability of potassium in the reaction medium facilitates the formation of this tunneled architecture. In addition, K^+^ ions play an electronic role: they provide charge compensation by balancing the negative charge arising from mixed-valence manganese ions (Mn^3+^/Mn^4+^); and they enhance thermodynamic stability through strong electrostatic interactions with the surrounding MnO_6_ framework, which increases the decomposition temperature.

**Fig. 3:**
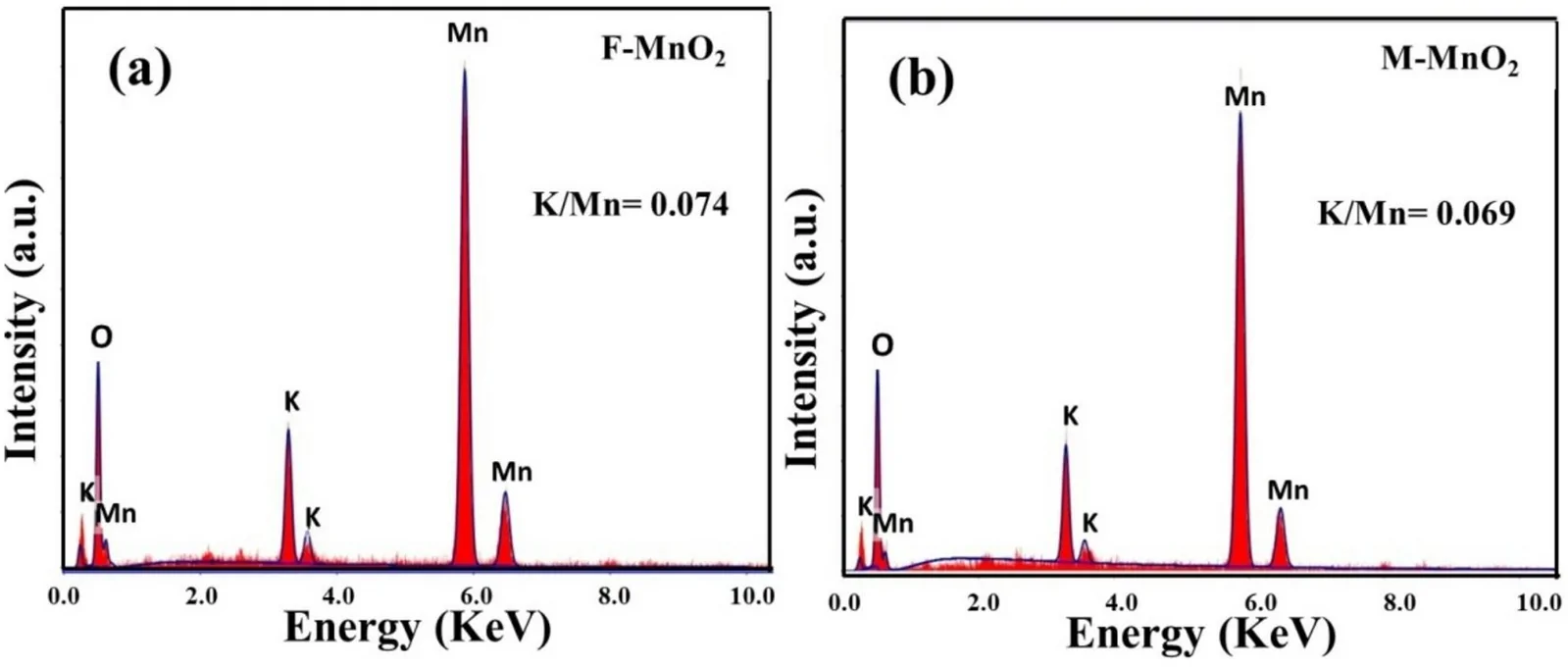
EDX spectra of green-synthesized MnO₂ using (**a**) female (F) and (**b**) male (M) Jojoba plant extracts.

To verify the elemental composition of the synthesized nanostructures, energy-dispersive X-ray spectroscopy (EDS) was performed (Fig. 3a, b).The analysis confirmed a predominant presence of manganese and oxygen, along with trace amounts of potassium, originating from the KMnO₄precursor^60^.The Mn content is 68 wt% for M-MnO_2_ and 76 wt% for F-MnO_2_. These results align with previous findings by Wang et al.^61^, who reported that KMnO₄ not only promotes the formation of Mn⁴⁺-rich species but also influences the resulting MnO₂ morphology. Quantitative EDS analysis revealed K/Mn atomic ratios of approximately 7.4% for the F-MnO₂ sample and 6.9% for M-MnO₂, indicating successful incorporation of potassium into the tunnel framework. Although present in low concentrations, K⁺ ions play a crucial role in stabilizing the α-MnO₂ structure and can enhance its electronic conductivityand influence surface properties, which are crucial for catalytic and antimicrobial applications^59,62^.The slightly higher K/Mn atomic ratio measured for F-MnO_2_ relative to M-MnO_2_ is consistent with, and provides a compositional basis for, its higher crystallinity and larger crystallite size of F-MnO_2_ relative to M-MnO_2_, discussed below, and underlies the charge-balance interpretation of the trace β-MnO_2_ signature detected in M-MnO_2_by Raman spectroscopy (Sect. 3.3).

X-ray diffraction analysis confirmed that both M-MnO_2_ and F-MnO_2_ crystallize in the cryptomelane-type α-MnO_2_ phase, which adopts a body-centered tetragonal unit cell (space group I4/m) characterized by (2 × 2) tunnels that accommodate K⁺ ions. These potassium ions, introduced during the redox synthesis using KMnO_4_ or K_2_Mn_2_O_8_, lead to a general formula of KₓMn_8_O_16_, where x represents the tunnel occupancy and typically remains below 0.5 per site in the lattice^63^.

To independently verify the potassium content determined by EDS, thermal analysis was employed. The decomposition temperature of MnO2 to Mn_2_O_3_ is known to correlate strongly with the tunnel K⁺ concentration, allowing for indirect quantification through thermogravimetric analysis (TGA).

#### Thermal gravimetric analysis (TGA) and derivative thermogravimetric (DTG)

**Fig. 4:**
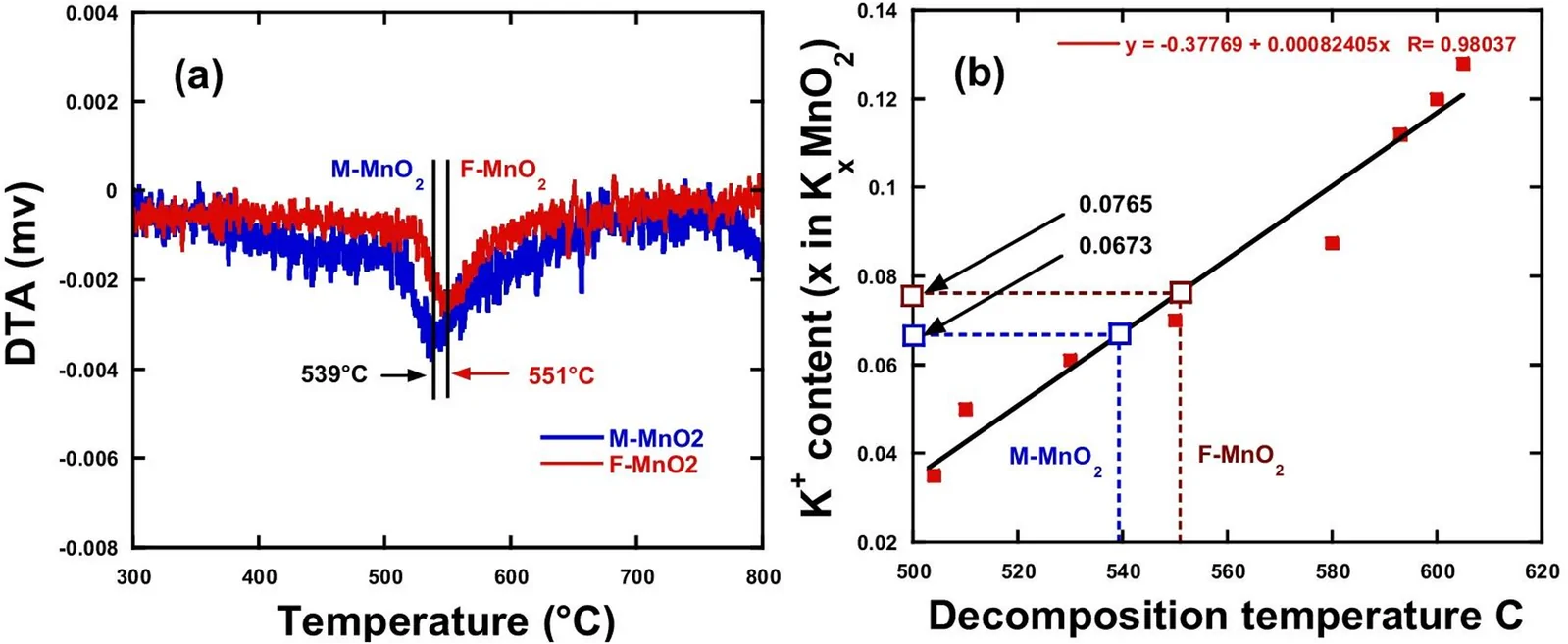
(**a**) Plots of the derivative weight loss vs. temperature showing the decomposition of MnO_2_ to Mn_2_O_3_ at T > 500 °C. (**b**) The concentration of K^+^ ions in the (2 × 2) tunnels of the α-K_x_MnO_2_ frameworks vs. the decomposition temperature. The linear relationship was derived from the experimental data based on DTG analyses previously reported in the literature^50,64–67^.

The derivative thermogravimetric (DTG) curves for both samples (Fig. 4a)show prominent decomposition peaks above 500 °C. By comparing these peak temperatures with previously reported DTG data^50,64–67^, a nearly linear correlation between the decomposition temperature and potassium (K⁺) content was identified (Fig. 4b). Based on this correlation, the observed decomposition temperatures 539 °C for M-MnO₂ and 551 °C for F-MnO₂ correspond to estimated potassium contents of x = 0.0673 and x = 0.0765, respectively. These values are in close agreement with the elemental ratios determined by EDS, supporting the reliability of both compositional and thermal analyses. This consistency confirms the effective incorporation of potassium into the tunnel structure of the biosynthesized α-MnO₂nanomaterials.

Consistent with the tunnel-stabilizing role of K^+^ described above, this higher estimated K^+^ content directly accounts for the higher decomposition temperature of F- relative to M-MnO_2_: greater tunnel occupancy requires more thermal energy to trigger collapse of the (2 × 2) framework and topotactic transformation of MnO_2_ to Mn_2_O_3_. This K^+^-content-decomposition temperature relationship is well established in the cryptomelane literature and is the basis for the indirect potassium quantification used in this study.

The combined results from XRD, EDS, and thermal analyses confirm that both M-MnO₂ and F-MnO₂ adopt the cryptomelane-type α-MnO₂ structure (KₓMn₈O₁₆), distinguished by its characteristic (2 × 2) tunnel framework capable of hosting potassium ions within the crystal lattice^68,69^. The incorporation of K⁺ ions into these tunnels contributes to the thermodynamic stabilization of the structure and significantly affects the surface properties of the materials factors that are essential in determining their reactivity and potential antimicrobial activity^70,71^.

The structural differences between the two samples, most notably the higher crystallinity and larger crystallite size of F-MnO₂ compared to M-MnO₂ combined with variations in potassium content, have a direct influence on their surface activity and catalytic behavior. These morphological and compositional features are closely associated with enhanced reactive oxygen species (ROS) generation and improved interactions with microbial cell membranes key mechanisms underlying the antimicrobial performance of manganese dioxide nanomaterials^51,72^. The synergistic interplay of an optimized tunnel structure, tailored crystallinity, and elevated surface reactivity highlights the potential of both materials as effective candidates for biomedical and environmental antimicrobial applications^73^.

**Fig. 5:**
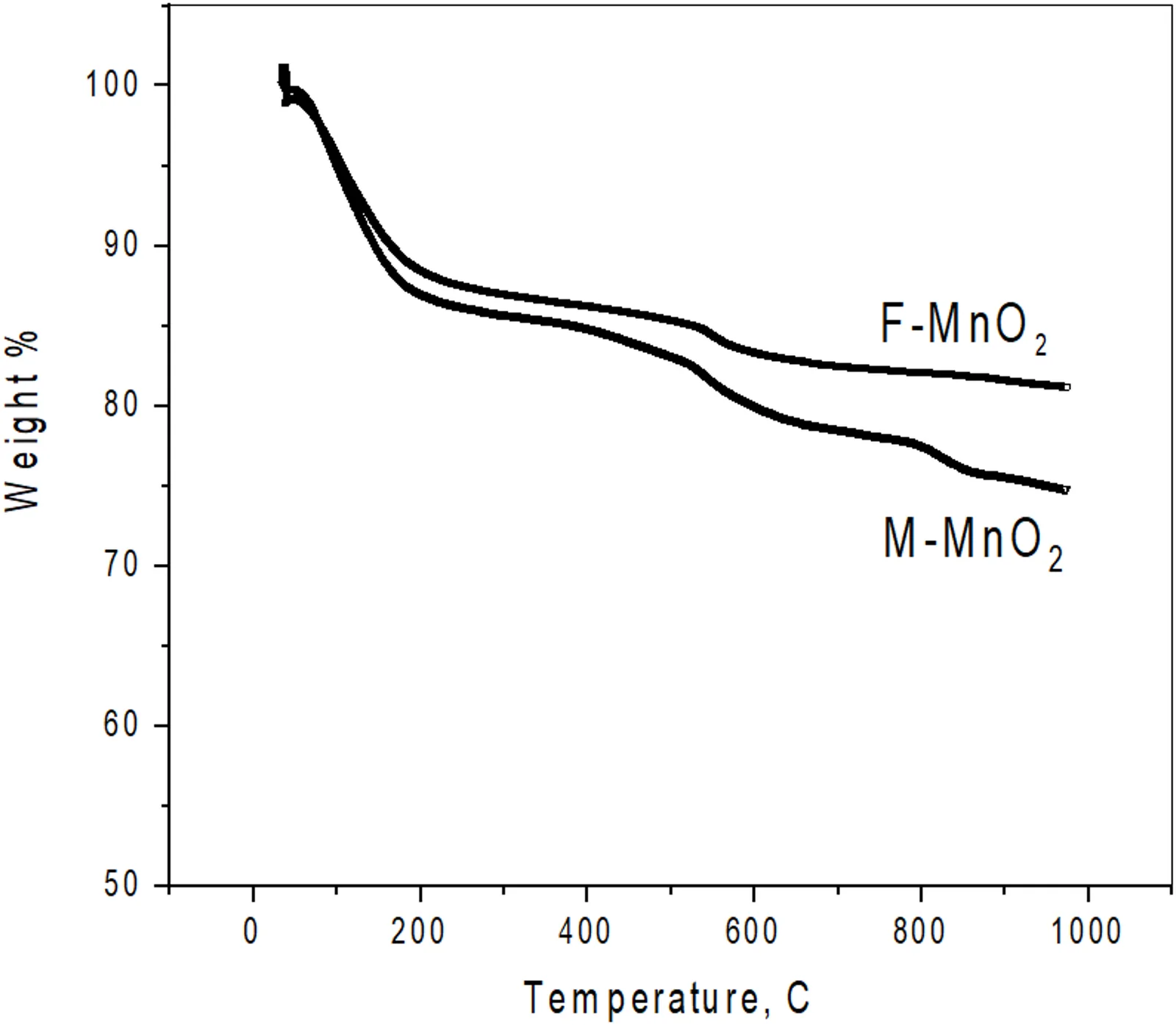
Thermogravimetric analysis of green-synthesized MnO_2_ using female (F-MnO_2_) and male (M-MnO_2_) Jojoba plant extracts.

Figure 5 throws more light on the thermal behaviors of both samples. It shows the thermal decomposition of two samples, M-MnO_2_and F-MnO_2_. Since the thermal behaviors are very close and seem to completely overlap with each other. So we will focus on the general trends, as they are representative of both samples. The TGA curve reveals weight loss in distinct stages as the temperature increases. A sharp drop in weight, from 99% to approx. 88%, occurs in this initial region. This significant weight loss is typically attributed to the desorption of physically adsorbed water (surface moisture) and the loss of loosely bound water (e.g., structural water, within the material). This stage confirms the samples are somewhat hydrated or absorb moisture from the atmosphere. The weight loss for M-MnO_2_ appears slightly more pronounced than for F-MnO_2_ in this initial region. Intermediate weight loss was noticed from 200 to 550 °C with gradual weight loss from ca. 88 to 84%. This loss may correspond to the decomposition of residual precursors (if any), the dehydroxylation (loss of tightly bound {OH} groups), or the further breakdown of structural components within the manganese oxide.For manganese dioxide there is loss of lattice oxygen or the thermal transformation from one oxide phase MnO_2_ to Mn_2_O_3_ below 600 °C and from Mn_2_O_3_ to Mn_3_O_4_ at higher temperature. Here, above approximately 600 °C and up to 1000 °C, the rate of weight loss significantly decreases, and the curves become relatively flat. This indicates that the remaining material has reached a stable state in the form of Mn_2_O_3_ until further expected decomposition to Mn3O4 as another thermally stable oxide.The final remaining weight percentages at 1000 °C are approximately 81% for F-MnO_2_ and slightly lower around 74% for M-MnO_2_. This remaining weight represents the mass of the final stable product. In Conclusion, F-MnO_2_ shows a slightly higher remaining weight throughout the analysis, particularly in the high-temperature region.

### Microstructural analyses

**Fig. 6:**
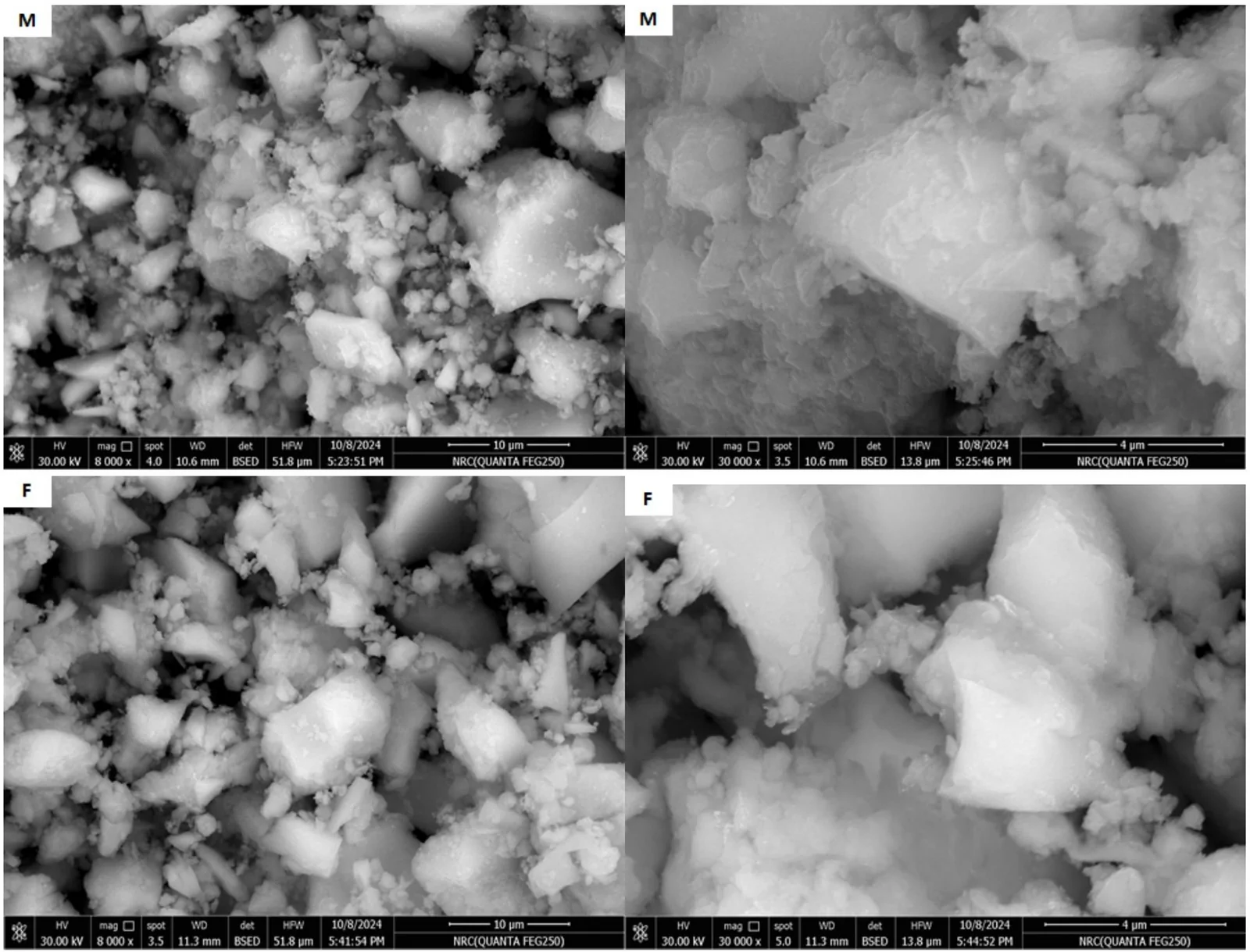
SEM of M-MnO_2_ and F-MnO_2_.

SEM analysis was performed to examine the morphological characteristics and surface structure of the synthesized samples in detail; the corresponding micrographs are presented in Fig. 6. The observations indicate that magnification significantly influences the apparent morphology of the nanoparticles by revealing features associated with nucleation and growth. At low magnification, both M-MnO_2_ and F-MnO_2_ samples exhibit non-uniform and heterogeneous α-MnO_2_ structures, attributed to particle adhesion and agglomeration. The samples appear as a mixture of three-dimensional, dandelion-like nanosized particles along with larger secondary agglomerates. In contrast, high-magnification images reveal that the powders consist of flake-like particles arranged in densely stacked, sheet-like structures. The flakes within the F-MnO_2_ sample appear more fused, which may be related to the nanocrystalline nature of the bio-synthesized MnO_2_. Notably, the flake morphology provides a high surface-area-to-volume ratio, thereby enhancing the material’s sensitivity and surface reactivity which in role affect the antimicrobial applications^60^. Due to the pronounced agglomeration and accumulation of particles, accurate determination of individual particle size is challenging. These morphological observations will be further validated in the TEM analysis section.

It should be noted that the aggregation evident in the SEM micrographs has two opposing consequences for the interpretation of the antimicrobial results. On one hand, agglomeration reduces the externally accessible surface area relative to what would be expected from fully dispersed primary particles of the same size, which likely means that the intrinsic (per-unit-area) reactivity of the nanosheets is understated by the bulk inhibition-zone and CFU-reduction data reported here. On the other hand, the flake-like, loosely stacked secondary architecture observed for both samples preserve a substantial fraction of interparticle void space and edge sites, so that the antimicrobial activity measured should be regarded as a property of this specific agglomerated morphology rather than of isolated single nanoparticles. This distinction is important because it means the higher activity of F-MnO_2_ cannot be attributed to smaller free-particle size alone, but instead reflects the combined effect of higher crystallinity, larger crystallite size, and greater potassium content described in Sect. 3.1–3.3.

**Fig. 7:**
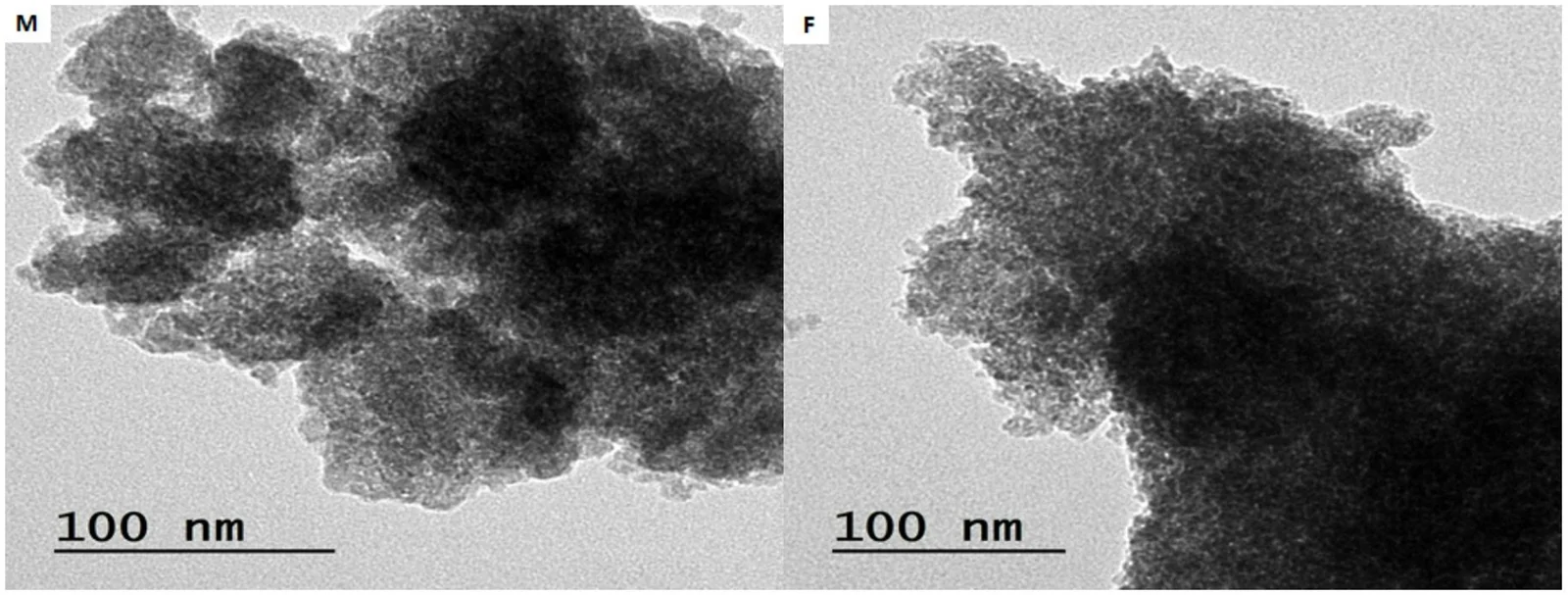
TEM of M-MnO_2_and F-MnO_2_.

According to the Transmission Electron Microscope (TEM) images (Fig. 7), we can notice that both samples have very fine interconnected and fused nanoparticles confirming the results obtained from the SEM analysis. The poor crystallization may be the reason behind this accumulation due to the difficulty of the growth of the particles during the synthesis after their nucleation. The size of the nanoparticles is in the nanometer scale that looks the same for the two samples with about 8 nm. This agreed with the calculated values in XRD section. These nanoparticles are expected to have high surface area which will affect positively on the antibacterial performance.

### Raman spectroscopic analysis

**Fig. 8:**
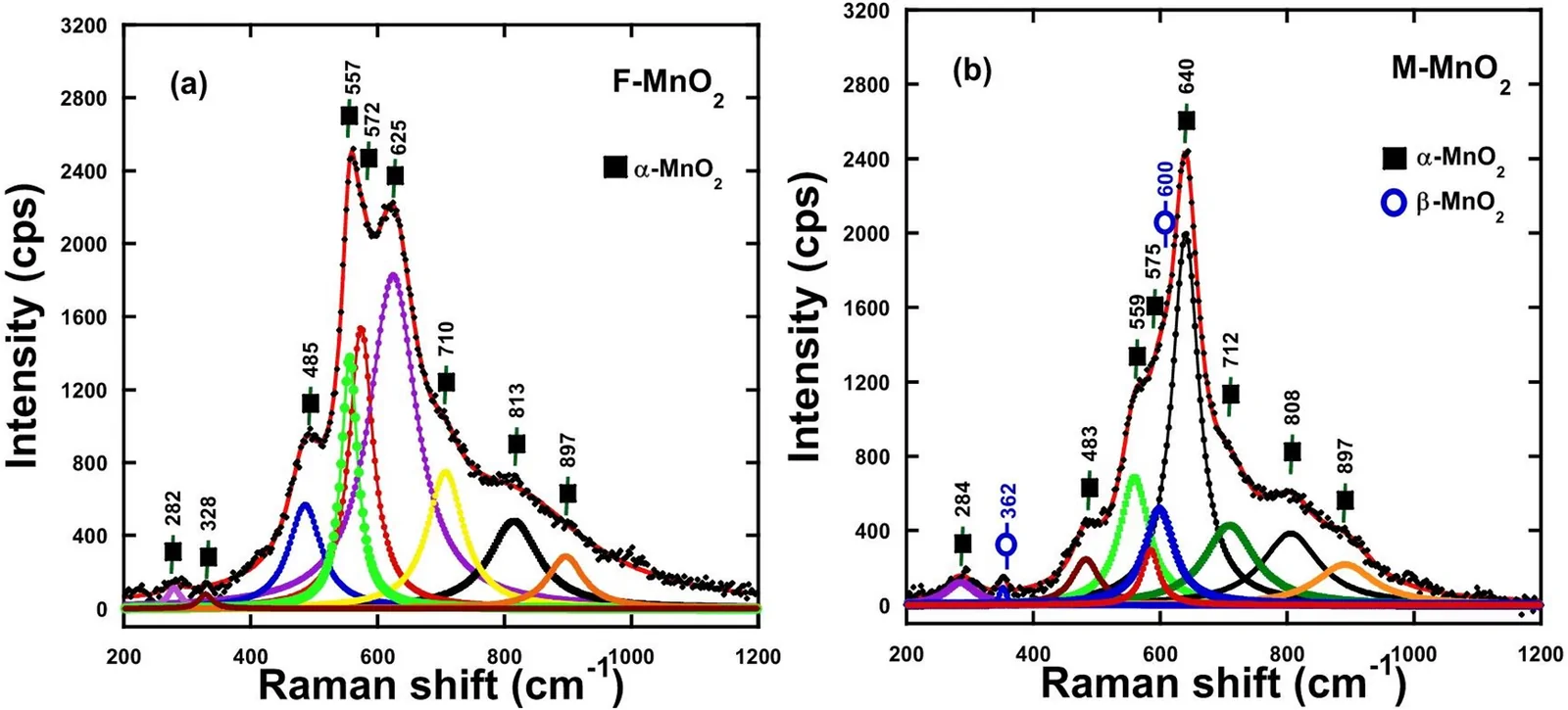
Raman Spectra of MnO_2_ nanoparticles synthesized from female (a) and male (b) jojoba plant extracts.

Raman spectroscopy, a non-destructive analytical technique, was employed to examine the structural properties of MnO_2_ nanoparticles synthesized through a green method using aqueous leaf extracts from male and female jojoba plants (Fig. 8a, b). All spectra were baseline-corrected (linear/polynomial fluorescence background subtraction) and intensity-normalized to the strongest band prior to peak assignment and comparison between samples, and weak bands (indicated by an asterisk in Table 2) were confirmed only after this correction.The technique is especially sensitive to ordered and short-range atomic arrangements, providing valuable complementary information to XRD, particularly for disordered or partially amorphous materials^74^. It offers critical insights into crystallinity, lattice distortions, and polymorphic phase content, where the nature of the bio-reducing plant extracts plays a significant role in determining the final phase and structural integrity of the nanoparticles.

Due to their polycrystalline nature, the Raman spectra of the samples do not display all theoretically predicted active modes (6Ag + 6Bg + 3Eg) for the ideal body-centered tetragonal MnO_2_ structure^75^. However, several distinct bands were observed, allowing comparative analysis of structural characteristics between the two samples.

The Raman spectrum of MnO_2_ synthesized with female jojoba extract (F-MnO_2_) exhibits sharp, well-resolved peaks at 283, 328, 485, 557, 572, 625, 710, 808 and 897 cm^− 1^ (Fig. 8; Table 2). Inductive of a highly crystalline material dominated by α- MnO_2_ phase. In contrast, the spectrum of MnO_2_ derived from male jojoba extract (M-MnO_2_) shows broader and less intense bands, indicating reduce crystallinity and the likely coexistence of multiple polymorphs.

The observed Raman bands can be grouped into three distinct spectral regions:

1. Low-frequency region (280–300 cm^− 1^)

Both samples exhibit a peak near 283 ± 1 cm^− 1^, attributed to Mn–O bending vibrations within distorted MnO_6_octahedra ^76–78^. A slight blue shift in this band for the M-MnO_2_ sample may reflect differences in Mn oxidation state, Mn–O bond strength, or local disorder. Additionally, a prominent band at 328 cm^− 1^ in F-MnO_2_, only weakly present in M-MnO_2_, corresponds to ordered lattice vibrational modes in α- MnO_2_ structure^79^. Notably, a weak band at 362 cm^− 1^, characteristic of β-MnO_2_ (pyrolusite phase), was observed exclusively in the M-MnO_2_ sample^80^, indicating the presence of mixed phases.The absence of this phase in the XRD pattern is likely due to its low abundance or poor crystallinity, underscoring the greater sensitivity of Raman spectroscopy in detecting subtle structural variations and mixed-phase compositions.

2. Mid-frequency region (480–650 cm^− 1^)

This region showed the most pronounced differences. The F-MnO_2_ exhibited sharp peaks at 485, 557, 572, and 625 cm^− 1^, consistent with α-MnO_2_ vibrational modes^81,82^. In contrast, M- MnO_2_ displayed broader features at 483, 559, 600, and 640 cm^− 1^. The shared band around 558 ± 1 cm^− 1^, attributed to MnO_6_ symmetric, is a signature of α- MnO_2.,_ However, the additional peaks in M-MnO_2_ at 600 and 640 cm^− 1^, alongside the 362 cm^− 1^ band, support the coexistence of β-MnO_2_ with the dominant α-MnO_2_ phase^80^.

These findingsare consistent with Yin et al.^83^, who demonstrated that the balance between K⁺ and H⁺ ions during synthesisplays a critical role in determines the resulting MnO_2_polymorph: a higher K⁺/H⁺ ratio favors the formation of tunnel-structured α-MnO_2_, as observed for the F-MnO_2_ sample, whereas relatively more acidic conditions promote the formation of β-MnO_2_, consistent with the mixed-phase character of M-MnO_2_. This is supported by the EDX-measured K/Mn atomic ratios reported in Sect.  3.1, which were slightly lower for M-MnO_2_ (6.9%) than for F-MnO_2_ (7.4%), indicating comparatively reduced K^+^ incorporation into the M-MnO_2_ tunnel framework during synthesis. Because charge neutrality within the (2 × 2) cryptomelane tunnel requires the co-occupying K^+^ and H⁺/H_3_O⁺ species to jointly compensate the octahedral-framework charge, this measurably lower K^+^ content corresponds, by charge balance, to a relatively higher effective proton occupancy of the tunnel sites in M-MnO_2_, i.e., a lower effective K⁺/H⁺ ratio. Per the mechanism of Yin et al.^83^, this lower K⁺/H⁺ ratio favors the nucleation of trace β-MnO_2_ domains alongside the dominant α-MnO_2_ phase, consistent with the minor M-MnO_2_. While male and female jojoba extracts are independently reported to differ in antioxidant and phytochemical profile^29,44,45^, the present data do not establish which specific extract components, or in which direction, derive this difference in K^+^ incorporation; we therefore ground this explanation in the measured EDX K/Mn ratio and the resulting charge-balance argument, rather than in an assumed direction of phytochemical reducing strength. This interpretation also explains the difference between Raman and XRD results. The absence of β-MnO_2_ reflections in the XRD patterns is attributed to its low abundance and/or poor crystallinity, placing it below the XED detection limit, whereas, Raman spectroscopy is greater sensitive to local bonding environments, short-range structural order and minor or poorly crystalline domains, thereby enabling the detection of trace β-MnO_2_that remains undetectable by XRD.

3. High-frequency region (700–900 cm^− 1^)

Raman bands in this range, (~ 710, ~ 810, and ~ 897 cm^− 1^) corresponding to antisymmetricMn–O stretching and overtone vibrations typical of tunnel-structured α- and β-MnO_2_^84,85^. These peaks were sharper and more defined in F-MnO_2_, reflecting crystallinity and phase purity. Conversely, the broader and less resolved peaks in M-MnO_2_ point to structure disordered and mixed-phase composition.

The structural, compositional, and spectroscopic differences observed between F-MnO_2_ and M-MnO_2_ highlight the critical influence of the biological reducing agent^86^. Female jojoba extracts, rich in phenolic and flavonoid compounds^55,87,88^, promote uniform nucleation and growth of the α-MnO_2_ phase, resulting in higher crystallinity. Male extracts, with a distinct phytochemical makeup, tend to favor the formation of mixed MnO_2_ polymorphs. These findings underscore the importance of phytochemical composition in green nanomaterial synthesis and provide a basis for tuning material properties through strategic choice of biological precursors.

**Table 2:** Raman band assignments of MnO_2_ synthesized using male and female jojoba plant extracts.

Raman shift (cm⁻¹)	F-MnO_2_ (female)	M-MnO_2_ (male)	Phase assignment	Interpretation
~ 282–284	✓	✓	α-MnO_2_	Lattice vibration / translational mode
328	✓	✓*	α-MnO_2_	Lattice vibrational mode (well-ordered structure)
362	✘	✓*	β-MnO_2_	Characteristic of β-MnO_2_ phase
483–485	✓	✓	α-MnO_2_	Mn–O bending vibration
557–559	✓	✓	α-MnO_2_	Intermediate Mn–O stretching in MnO_2_ octahedra
572–575	✓	✓	α-MnO_2_	Symmetric Mn–O stretching
600	✘	✓	β-MnO_2_	Mn–O stretching vibration of β-MnO_2_ (pyrolusite)
625	✓	✘	α-MnO_2_	Asymmetric stretching vibration
640	✘	✓	β-MnO_2_	Mn–O stretching in pyrolusite (secondary band)
710–712	✓	✓	α-MnO_2_	O–Mn–O bending / overtone
808–813	✓	✓	α-MnO_2_	Overtones or lattice combination
897	✓	✓	α-MnO_2_	High-frequency overtone (Mn–O bonding)

✗ = Not observed; ✓ = Observed. * = Weak intensity, detectable after baseline correction.

### The antimicrobial activity of the prepared samples

**Fig. 9:**
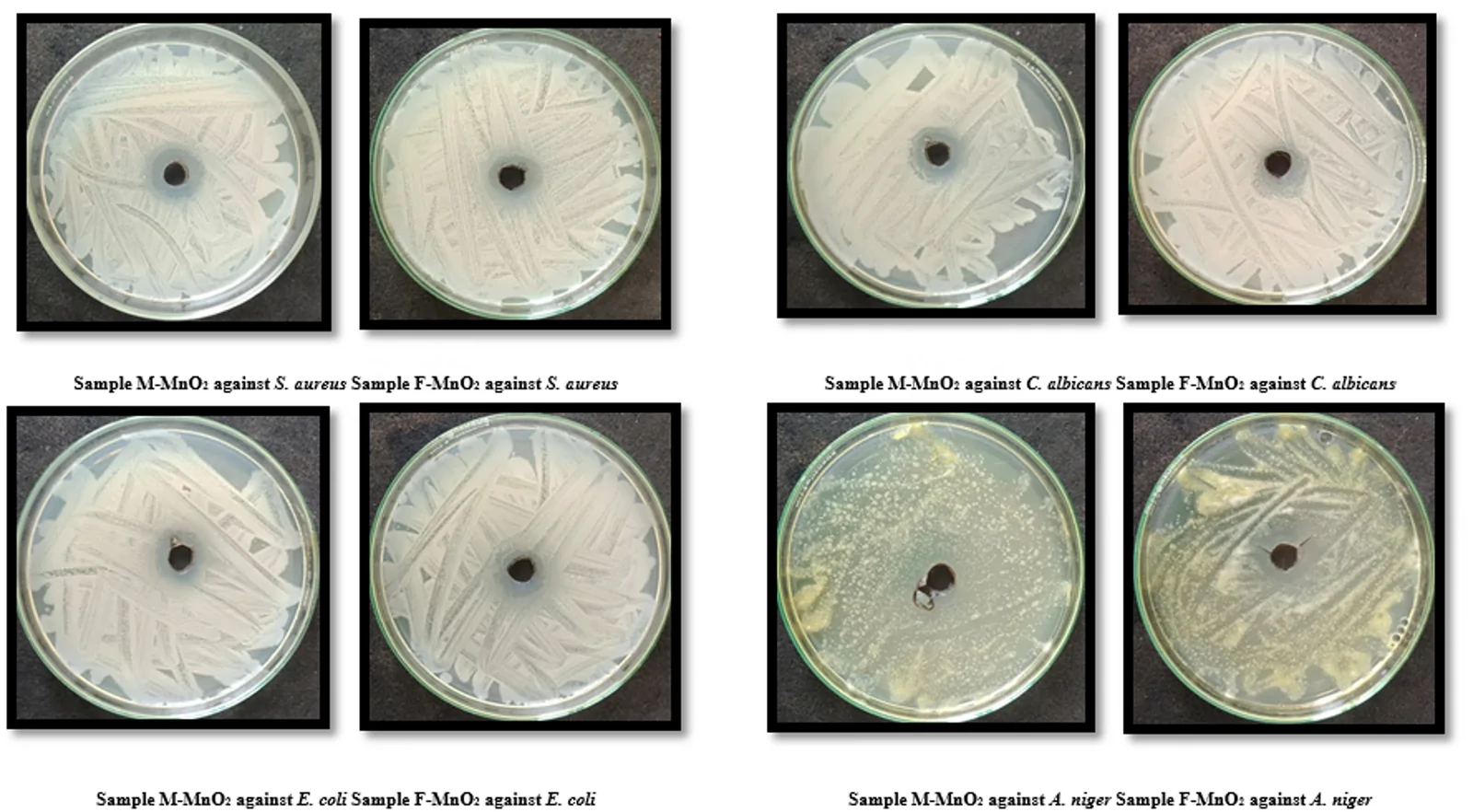
The antimicrobial activity of the as synthesized compounds (samples M-MnO_2_ and F-MnO_2_) against different groups of microbial strains including G + ve bacterium (*S. aureus*), G-ve bacterium (*E. coli*), yeast (*C. albicans*) and the filamentous fungus (*A. niger*) by the cup diffusion agar method.

**Fig. 10:**
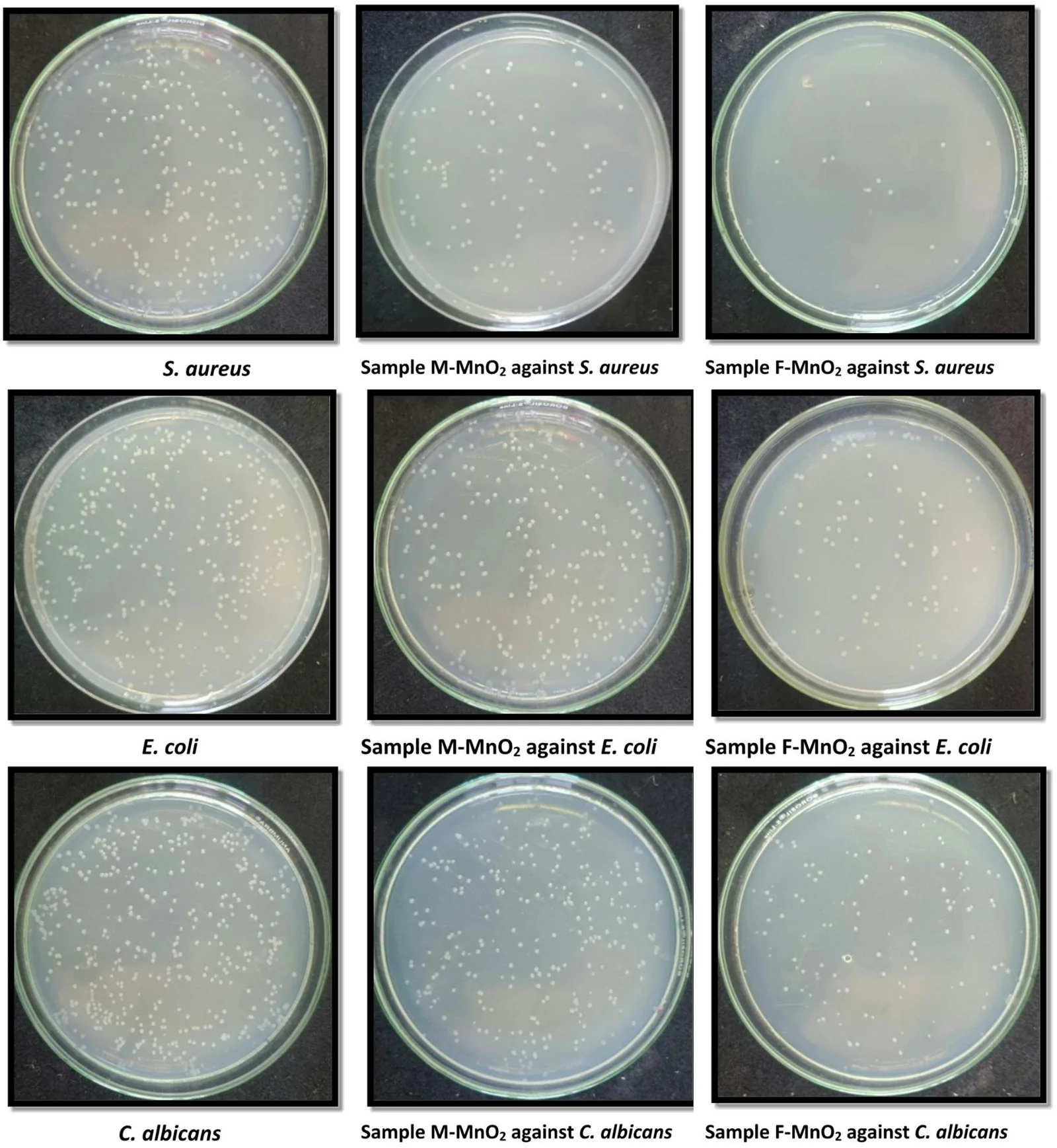
The antimicrobial activity of the synthesized compounds (samples M-MnO_2_ and F-MnO_2_) against different groups of microbial strains including G + ve bacterium (*S. aureus*), G-ve bacterium (*E. coli*) and yeast (*C. albicans*) by the colony forming unit (CFU) technique against selected microbial strains.

The cup diffusion assay offered a clear visual and spatial indication of antimicrobial activity, whereas the CFU assay provided a quantitative assessment of microbial growth inhibition. Together, these complementary methods enabled a robust and comprehensive evaluation of the antimicrobial efficacy of the test compounds across a diverse range of microorganisms. The antimicrobial activity of the synthesized compounds was tested against different microbial strains including G + ve bacterium (*S. aureus*), G-ve bacterium (*E. coli*), yeast (*C. albicans*) and the filamentous fungus (*A. niger*). Results in Table 3; Fig. 9 indicated that compound M-MnO_2_ exhibited antimicrobial activities against *S. aureus*, *E. coli*, *C. albicans* and *A. niger* with inhibition zones of 17, 16, 17 and 18 mm (respectively). On the other hand, compound F-MnO_2_ showed inhibition zones of 19, 17, 20 and 25 mm against the same test strains (respectively), consistent with the strong antibacterial potential reported for other green-synthesized doped nanoparticles from plant extracts^89,90^. Moreover, the antimicrobial activities of the previously mentioned compounds (M-MnO_2_ and F-MnO_2_) were tested against *S. aureus*, *E. coli* and *C. albicans* using the counting method (CFU/ml). Results in Table 4 and Fig. 10 revealed that M-MnO_2_ and F-MnO_2_ had growth reduction (R%) of 57.39 and 94.78% against S. aureus (respectively). The two compounds exhibited R% of 21.25 and71.08 against *E. coli* (respectively). M-MnO_2_ and F-MnO_2_ showed R% of 36.39 and 92.41% against *C. albicans* (respectively). Results concluded that F-MnO_2_ surpasses M-MnO_2_ as antimicrobial agent.The superior antimicrobial performance of F-MnO_2_ relative to M-MnO_2_ can be tentatively attributed to differences in crystallinity, phase composition, and surface reactivity established through the Raman and XRD analyses above. The dominant, highly crystalline α-MnO_2_ phase in F-MnO_2_ is expected to support more efficient Mn³⁺/Mn⁴⁺ redox cycling at the particle surface, a process associated with generation of reactive oxygen species (ROS) capable of damaging bacterial membranes, proteins, and DNA. In contrast, the mixed α-/β-MnO_2_ phase composition and reduced crystallinity of M-MnO_2_ may correspond to a lower density of redox-active surface sites, potentially limiting ROS-mediated antibacterial action. Previous studies on male and female jojoba plants have reported sex-based differences in antioxidant compound levels and physiological profiles^91,92^, which may plausibly extend to differences in phenolic and flavonoid content between the extracts used in this study; such compounds, if present, could contribute residual surface-bound bioactivity capable of independently interacting with bacterial membranes, complementing the nanoparticle-mediated mechanism. However, we did not perform direct phytochemical quantification (e.g., total phenolic content, total flavonoid content) in this study, and this remains a hypothesis rather than a confirmed mechanism. We note that these mechanistic explanations remain inferential, as they are not yet supported by direct ROS quantification, Mn ion release measurements, or phytochemical assays, and we identify this as an important direction for future work.

**Table 3:** Antimicrobial activity (zone of inhibition, mm; mean ± SD, *n* = 3) of the as-synthesized samples against different test microorganisms.

Sample Name	Clear zone(mm)			
	*S. aureus*	*E. coli*	*C. albicans*	*A. niger*
M-MNO_2_	17.3 ± 0.6	16.3 ± 1.5	16.7 ± 0.6	18.3 ± 0.6
F-MNO_2_	17.7 ± 1.5	18.0 ± 1.0	19.0 ± 1.0	25.3 ± 0.6
Neomycin	25.0 ± 1.0	26.7 ± 0.6	23.0 ± 1.0	0 ± 0
Cyclohexamide	0 ± 0	0 ± 0	0 ± 0	28.0 ± 1.0

**Table 4:** The CFU value at different dilutions of as synthesized samples against different test microbes.

Microbe name	Samples	Colony forming units (CFU) (x10^− 3^)	
		No. of colonies	Reduction (*R*%)
*S. aureus*	*S. aureus*	230	–
	M-MnO_2_	98	57.39
	F-MnO_2_	120	94.78
*E. coli*	*E. coli*	287	–
	M-MnO_2_	226	21.25
	F-MnO_2_	83	71.08
*C. albicans*	*C. albicans*	327	–
	M-MnO_2_	208	36.39
	F-MnO_2_	108	92.41

## Conclusion

This research investigates the potential of jojoba plant as a natural antioxidant source abundant in Egypt. The study focused on the leaf extracts of male and female jojoba plants. The XRD results show the higher crystallinity with larger crystallite size of F-MnO₂ (25 nm) compared to M-MnO₂ (15 nm). The EDX analysis reveals K/Mn atomic ratios of approximately 7.4% for the F-MnO_2_ sample and 6.9% for M-MnO_2_.The thermal analysisdemonstrated the thermal stability of both samples at the high temperature region which candidates them for the antimicrobial applications.The combined analyses via XRD, EDS, and thermal methods unequivocally demonstrate that both M-MnO₂ and F-MnO₂ exhibit the cryptomelane-type α-MnO₂ structure characterized by a (2 × 2) tunnel framework. The presence of potassium ions within these tunnels not only stabilizes the crystal structure thermodynamically but also plays a crucial role in modulating the surface properties. These structural and compositional features are key determinants of the materials’ reactivity and their promising potential for antimicrobial applications.M-MnO_2_ exhibited antimicrobial activities against *S. aureus*, *E. coli*, *C. albicans* and *A. niger* with inhibition zones of 17, 16, 17 and 18 mm (respectively). On the other hand, compound F-MnO_2_ showed inhibition zones of 19, 17, 20 and 25 mm against the same test strains (respectively). Antimicrobial activities of the previously mentioned compounds (M-MnO_2_ and F-MnO_2_) were tested against *S. aureus*, *E. coli* and *C. albicans* using the counting method (CFU/ml). The results revealed that M-MnO_2_ and F-MnO_2_ had growth reduction (R%) of 57.39 and 94.78% against S. aureus (respectively). The two compounds exhibited R% of 21.25 and71.08 against *E. coli* (respectively). M-MnO_2_ and F-MnO_2_ showed R% of 36.39 and 92.41% against *C. albicans* (respectively). Results concluded that F-MnO_2_ surpasses M-MnO_2_ as antimicrobial agent.

A limitation of the present study is that antimicrobial activity was assessed using disk diffusion and CFU reduction assays only, without determination of MIC, MBC, or MFC values, and without independent characterization of nanoparticle solubility/dispersion behavior in the assay medium. Antifungal activity against *A. niger* was also evaluated at a single time point, so the durability of this effect (fungistatic versus fungicidal) remains to be established. Building on these findings, future work will therefore (i) determine MIC, MBC, and MFC values against a wider microbial panel, incorporating broth microdilution assays, extended time-course monitoring, a standard antibiotic/antifungal positive control, a DMSO-only negative control, and full triplicate statistics; (ii) quantify total phenolic and flavonoid content of the male and female extracts to place the observed structural differences on a compositional basis; (iii) perform Rietveld refinement and BET surface-area analysis to obtain quantitative crystallinity and area-normalized antimicrobial metrics; (iv) monitor synthesis pH in situ to directly correlate reaction conditions with the α-/β-MnO_2_ phase selectivity identified by Raman spectroscopy; and (v) conduct ROS quantification and metal ion release profiling to provide a more mechanistically grounded assessment of the antimicrobial differences between F-MnO_2_ and M-MnO_2_.

## Data Availability

The datasets generated and/or analyzed during the current study are available from the corresponding author on reasonable request.
